# Differential *in vivo* expression of mycobacterial antigens in *Mycobacterium tuberculosis*infected lungs and lymph node tissues

**DOI:** 10.1186/1471-2334-14-535

**Published:** 2014-10-03

**Authors:** Tehmina Mustafa, Nils Anders Leversen, Lisbet Sviland, Harald Gotten Wiker

**Affiliations:** Centre for International Health, Department of Global Public Health and Primary Care, University of Bergen, Bergen, Norway; Department of Thoracic Medicine, Haukeland University Hospital, Bergen, Norway; Department of Clinical Science, University of Bergen, Bergen, Norway; Section for Pathology, Department of Clinical Medicine, University of Bergen, Bergen, Norway; Department of Pathology, Haukeland University Hospital, Bergen, Norway; Department of Microbiology, Haukeland University Hospital, Bergen, Norway

**Keywords:** Mycobacterium tuberculosis, Intracellular infection, Pathology, Granuloma, MPT32, MPT44, MPT46, MPT51, MPT53, MTP59, MPT63, MPT64, Mce1A, Hsp65, MPT57

## Abstract

**Background:**

The clinical course of tuberculosis (TB) infection, bacterial load and the morphology of lesions vary between pulmonary and extrapulmonary TB. Antigens expressed in abundance during infection could represent relevant antigens in the development of diagnostic tools, but little is known about the *in vivo* expression of various *M. tuberculosis* antigens in different clinical manifestations. The aim of this study was to study the differences in the presence of major secreted as well as somatic mycobacterial antigens in host tissues during advanced rapidly progressing and fatal pulmonary disease with mainly pneumonic infiltrates and high bacterial load, and to compare this to the presence of the same antigens in TB lymphadenitis cases, which is mainly chronic and self-limiting disease with organised granulomas and lower bacterial load.

**Methods:**

Human pulmonary (n = 3) and lymph node (n = 17) TB biopsies, and non-TB controls (n = 12) were studied. Ziehl-Neelsen stain, nested PCR 1S6110 and immunohistochemistry were performed. Major secreted (MPT32, MPT44, MPT46, MPT51, MPT53, MPT59, MPT63, and MPT64) and somatic mycobacterial antigens (Mce1A, Hsp65, and MPT57) were detected by using rabbit polyclonal antibodies.

**Results:**

Plenty of bacilli were detectable with Ziehl-Neelsen stain in the lung biopsies while no bacilli were detected in the lymph node biopsies. All the cases were shown to be positive by PCR. Both secretory and somatic antigens were expressed in abundance in pulmonary infiltrates, while primarily somatic antigens were detected in the lymphadenitis cases. Of the secreted antigens, only MPT64 was consistently detected in both cases, indicating a preferential accumulation of this antigen within the inflammatory cells, even if the cells of the granuloma can efficiently restrict bacterial growth and clear away the secreted antigens.

**Conclusions:**

This study shows that major secreted mycobacterial antigens were found in high amounts in advanced pulmonary lesions without proper granuloma formation, while their level of staining was very low, or absent, in the lymph node TB lesions with organised granulomas and very low bacillary load, with one exception of MPT64, suggesting its role in the persistence of chronic infection. These findings have implication for development of new diagnostic tools.

**Electronic supplementary material:**

The online version of this article (doi:10.1186/1471-2334-14-535) contains supplementary material, which is available to authorized users.

## Background

Infection with *Mycobacterium tuberculosis* represents a major disease burden globally. There are about 8,7 million new cases of tuberculosis (TB) worldwide and at least 1.4 million deaths per year [1]. Extra pulmonary TB accounts for approximately one-fifth of TB cases among immune-competent individuals and up to one-half in HIV-infected individuals [1–4]. Though any organ in the body can be involved, lymph nodes are the most common form of extra-pulmonary TB in both adults and children [5, 6]. Immune responses in TB differ between various disease sites and in various forms of disease [7, 8]. TB lymphadenitis is typically a self-contained, while pulmonary TB is usually a rapidly progressive and fatal disease without adequate chemotherapy.

In humans, granuloma formation in response to *M. tuberculosis* infection is essential for control of mycobacterial infections [9]. Paradoxically, granulomatous inflammation is also associated with the typical immunopathology, tissue damage and symptoms seen in TB. It is believed that mycobacterial antigens are continuously released into the infected tissue and could be responsible for the formation and persistence of lesions [10–13]. Antigens secreted into the extracellular environment by *M. tuberculosis* are thought to be immunodominant and to be involved in inducing protective immunity, and in this sense also considered to be the most important antigens during infection [14, 15]. Little is known about the identity of the *M. tuberculosis*-antigens that are expressed *in vivo* in the lesions. TB is a chronic and multi-organ disease, and the expression profile of antigens in the infected tissues may be different during various phases of infection and in various organs, making it important to study the differential *in vivo*-expressed antigens in various organs with different disease manifestation.

Previous work has identified and purified major secretory and somatic *M. tuberculosis* antigens to near homogeneity from the culture filtrates and bacterial sonicates from *M. tuberculosis* cultured in wholly synthetic liquid media [16–18]. Functional rabbit polyclonal antibodies have been generated against these antigens [17, 19, 20]. This collection of antibodies was used to study the *in situ M. tuberculosis* antigens expression in the infected host tissues. We have previously performed several studies on MPT64 for the development of immunohistochemical method using patient biopsies and aspirate samples to diagnose extrapulmonary TB [21–24]. In this study, we have focused on exploring the expression pattern of several additional major secreted (MPT32, MPT44, MPT46, MPT51, MPT53, MPT59, MPT63, and MPT64) and somatic mycobacterial antigens (Mce1A, Hsp65, and MPT57) and studied the differential expression in the host tissues during advanced rapidly progressing and fatal pulmonary disease with mainly pneumonic infiltrates, and compared this to the presence of the same antigens in TB lymphadenitis cases, which is mainly chronic and self-limiting disease with organised granulomas.

## Methods

### Study patients and controls

Seventeen cases of human tuberculous lymphadenitis and three cases of human pulmonary TB were studied. The patients had either pulmonary or lymph node TB. All the TB lymphadenitis cases had typical histological features of necrotic granulomas consistent with the diagnosis of TB. These were also confirmed as TB cases based on the combined clinical, microbiological, and histological criteria. The pulmonary TB cases were confirmed based on the histology and the detection of tuberculous bacilli from the lesions. Both types of cases were further confirmed to be caused by *M. tuberculosis* complex organisms by positive nested-PCR based on the amplification of IS6110 as described earlier [22]. Negative controls included 9 foreign-body granulomas of the skin, and 1 each from colon cancer, normal tonsillar tissue, and lung tissue from ischemic heart disease. Based on the clinical information, latent or active TB was excluded from the controls. The controls were further confirmed to lack *M.tuberculosis* infection by negative nested-PCR of IS6110, negative histology and negative ZN staining. Table 1 shows the baseline features of patients and controls.

**Table 1:** **Baseline features of the study patients and controls**

	TB cases	Controls
***Age group***		
Children (2–14 years)	6	0
Adults (18–86 years)	14	12
***Gender***		
Male	12	6
Female	8	6
***Origin of biopsies***		
Lung tissues from pulmonary TB with many AFBs	3	
Cervical lymph nodes	13	
Axillary lymph nodes	2	
Mesenteric lymph nodes	1	
Inguinal lymph nodes	1	
Foreign-body granulomas of the skin		9
Colon cancer		1
Normal tonsillar tissue		1
Lung tissues from autopsy of ischemic heart disease		1

### Ethics statement

Ethical clearance was obtained from the regional ethical committee of western Norway. The ethical committee provided exemption for the written informed consent statement for this study as the biopsies were obtained from a bio-bank consisting of already collected anonymised biomaterial for research purposes.

### Histology and immunohistochemistry

Parallel 5 μm thick sections from each specimen were stained with haematoxylin and eosin, and immunostaining using the EnVision + System-HRP kit (DakoCytomation Denmark A/S, Glostrup, Denmark) was done as described previously [22]. Briefly, after deparaffinisation and rehydration, the sections were exposed to microwave-antigen-retrieval using citrate buffer pH6.0 at 750 W for 10 min, and at 350 W for 15 min. The sections were cooled for 20 min at room temperature and then incubated with H_2_O_2_ solution for 5 min. Primary antibodies were then applied to the sections for 45 min, and subsequently incubated for 40 min with anti-rabbit immunoglobulin conjugated with dextran polymer and horseradish peroxidase (DakoCytomation Denmark A/S, Glostrup, Denmark). Visualisation was achieved using 3-amino-9-ethylcarbazol (AEC) containing H_2_O_2_ (DakoCytomation Denmark A/S, Glostrup, Denmark). Primary antibodies and dilutions used are shown in Table 2. Two negative controls were used; in the first, primary antibody was substituted with antibody diluent, and in the second, an irrelevant rabbit polyclonal antibody was used. In addition, pre-immune sera for some of the antibodies, and anti-GST anti-sera for anti-Mce1A, were used as controls.

**Table 2:** **Antibodies used in the immunohistochemical staining**

Antibody	Target antigen	Protein function	Dilution
***Secreted antigens***			
**Anti-MPT32**	**MPT32 (Apa, Rv1860)**	Unknown (Could mediate bacterial attachment to host cells)	1:500
**Anti-MPT44**	**MPT44 (Ag85A, fbpA, Rv3804c)**	Involved in cell wall mycoloylation. Responsible for the high affinity of mycobacteria to fibronectin. Possesses a mycolyltransferase activity required for the biogenesis of trehalose dimycolate (cord factor), a dominant structure necessary for maintaining cell wall integrity.	1:100
**Anti-MPT46**	**MPT46 (Thioredoxin TrxC, Rv3914)**	Participates in various redox reactions through the reversible oxidation of its active center dithiol, to a disulfide, & catalyzes dithiol-disulfide exchange reactions.	1:500
**Anti-MPT51**	**MPT51 (Ag85D, fbpD, Rv3803c)/TB22.2**	Same as MPT44	1:500
**Anti-MPT53**	**MPT53 (DsbE, Rv2878c)**	Unknown	1:500
**Anti-MPT59**	**MPT59 (Ag85B, fbpB, Rv1886c)**	Same as MPT44	1:100
**Anti-MPT63**	**MPT63 (Rv1926c)**	Unknown	1:500
**Anti-MPT64**	**MPT64 (Rv1980c)**	Unknown (Suggested to inhibit apoptosis of infected cells)	1:250
***Somatic antigens***			
**Anti-Mce1A**	**Mce1A (Rv0169)**	Thought to be involved in cell invasion (entry and survival)	1:2000
**Anti-Hsp65**	**Hsp65 (Ag82, GroEL2, Rv0440)**	Prevents misfolding and promotes the refolding and proper assembly of unfolded polypeptides generated under stress conditions.	1:500
**Anti-MPT57**	**MPT57 (GroES, Rv3418c)**	Binds to Cpn60 in the presence of Mg-ATP and suppresses the ATPase activity of the latter.	1:4000

### Antibodies

In-house antibodies were used in the study. These antibodies were raised by immunization of rabbits with antigens purified from *M. tuberculosis* as described earlier [17, 20]. Briefly, *M. tuberculosis* H37Rv (ATCC 27294), obtained from the National Institute of Health, Tokyo, Japan, was grown in wholly synthetic Sauton medium. The bacilli were removed by centrifugation and the culture supernatant was concentrated by 80% ammonium sulphate precipitation. The bacilli were washed three times in phosphate-buffered saline and sonicated in a rosette cooling cell with a model Branson B12 Sonifier. The antigens were purified by combining gel filtration and ion-exchange methods as described previously [17]. Polyclonal rabbit antibodies were obtained by immunising with soluble purified antigen using a standard immunization procedure with Freunds incomplete adjuvant [25]. Table 2 summarises the antibodies used and their specificities.

### Evaluation of immunostaining

Number of stained cells and total nuclei were counted in the lesions using a × 40 ocular fitted with a 10 × 10 mm grid. One section from each individual case and three areas per section were counted. It was difficult to have exact counts as many positive cells were clustered together. Due to error in the counting of positive cells and large intra-observer variability, it was chosen to present data as semi-quantitative analysis instead of exact numbers/percentages, where “+++++” was used to describe lesions where ≥ 80% granuloma cells were positive, “++++” if about 50% granuloma cells were positive, “+++” if 10-25% granuloma cells were positive, “++” if 2-9% granuloma cells were positive, “+” if <2% granuloma cells were positive, and “-” if no positive cells were detected. A qualitative description of the staining pattern was also done. A cut-off of positive cells to define a positive case was not used since extrapulmonary TB is usually paucibacillary. The staining in negative controls was instead discriminated as non-specific based on the quality and location of staining.

### Nested polymerase chain reaction for IS6110

Five to six, 8 μm sections from each paraffin embedded tissue blocks were collected in sample preparation tubes for nested PCR. Carry-over tissue contamination was prevented by cleaning the blade with 96% ethanol after sectioning each sample; negative controls were sectioned first, followed by test blocks and positive control blocks.

DNA extraction and nested PCR on paraffin sections were performed as described previously [22]. Briefly, following proteinase K digestion, bacterial genomic DNA was eluted in water using a MagAttract DNA mini M48 Kit (Qiagen,West Sussex, UK) on Biorobot M48 (Qiagen). A 123- base pair fragment from IS6110 was amplified using the following primers 5' CCTGCGAGCGTAGGCGTCGG 3' and 5' CTCGTCCAGCGCCGCTTCGG 3'. The product was subjected to a second round of PCR amplification using the primers 5' TTCGGACCACCAGCACCTAA 3' and 5' TCGGTGACAAAGGCCACGTA 3' to amplify a 92-base pair fragment. The PCR reaction mixture consisted of 5 μl eluted DNA, 25 μl of HotStarTaq master mix (Qiagen), 0.25 μl of each 100 μM primer stock solution, distilled water to make a final volume of 50 μl. For nested PCR, 1 μl of the first PCR product was used as template. The reaction cycle for the first PCR was- 94°C for 1 minute, 68°C for 1 minute, 72°C for 20 seconds for 45 cycles and for the nested PCR - 94°C for 1 minute, 58°C for 1 minute, 72°C for 20 seconds for 35 cycles. Both PCR’s had an initial heat activation step of 95°C for 15 minutes and a final extension of 72°C for 10 minutes. The amplified product was analyzed in a 3% agarose gel stained with ethidium bromide. Mycobacterial DNA, and positive PCR product were included as positive controls and an extraction control (with all the steps but without any tissue), a reaction tube with substitution of distilled water for the test template and a sample which previously yielded negative result on PCR were included as negative control in each PCR run.

## Results

### Morphology, acid-fast staining and PCR

All the lymph node biopsies had necrotic granulomatous inflammation consistent with TB. There were focal aggregates of epithelioid macrophages and multi-nucleated giant cells with a central necrotic area. The lung tissues showed widespread pneumonic infiltrates with chronic inflammatory cells, but lacking the necrotic granuloma morphology seen in lymphadenitis lesions. Plenty of bacilli were detectable with acid fast staining in the lung biopsies while no bacilli were detected by this method in the lymph node biopsies. All the cases were shown to be positive by nested PCR for IS6110, which is specific for *M. tuberculosis* complex organisms.

### Secreted mycobacterial antigens in lung tissues

**Figure 1:**
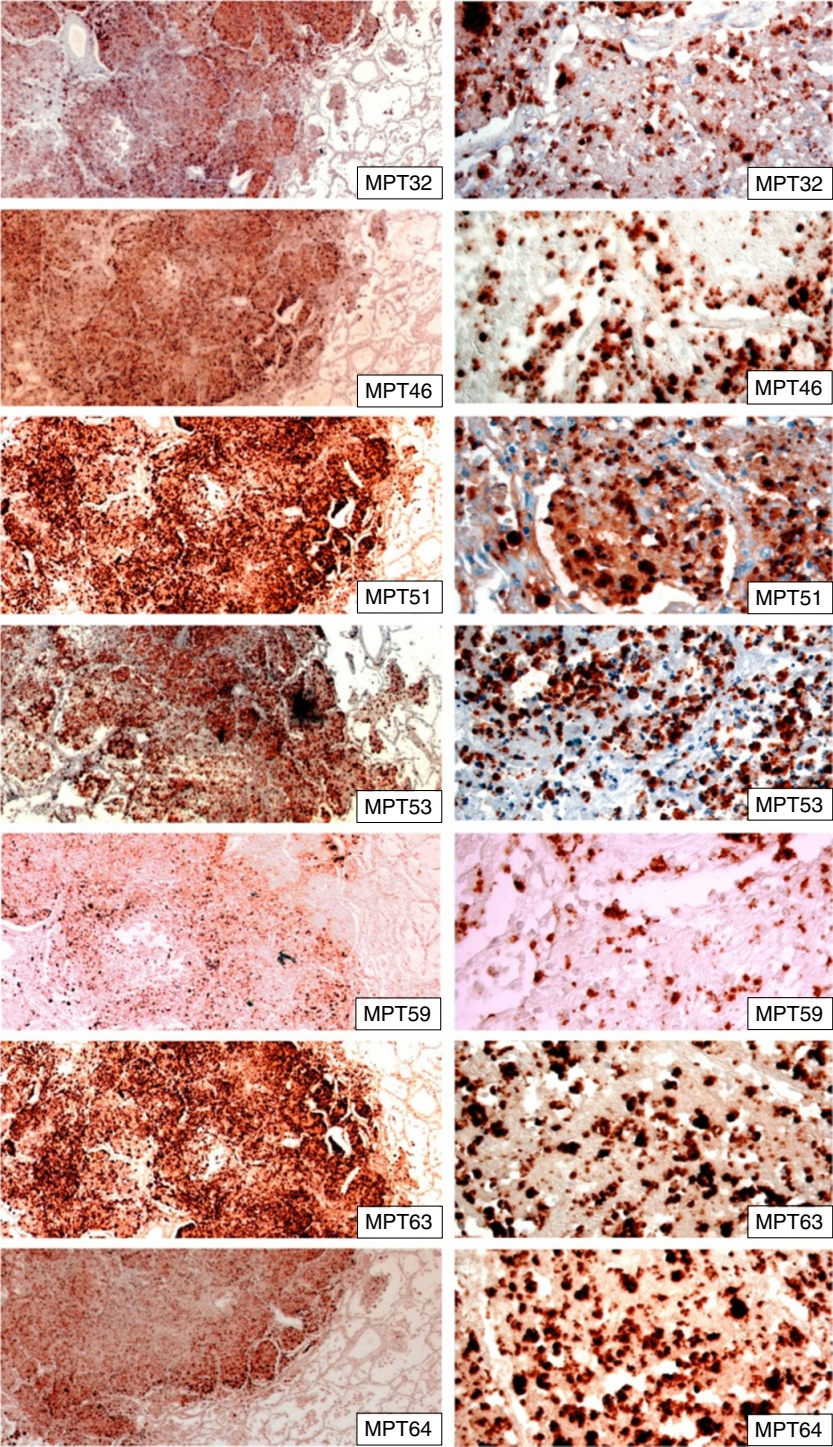
**Human pulmonary tissues infected with** ***M. tuberculosis*** **showing the staining pattern of secreted mycobacterial antigens in the lesions as detected by immunohistochemical staining.** The right column shows a higher magnification of corresponding pictures, and illustrates intracellular location of all antigens and the granular staining pattern. All antigens are expressed in abundance in the lesions. The staining pattern varies and MPT59 (Ag85B) was detected less frequently than other antigens. The staining is detected mainly in the inflammatory lesions, but a few macrophages in close proximity of the lesions were also stained by these antibodies.

The major secreted antigens; MPT32, MPT44 (Ag85A), MPT46, MPT51 (Ag85D), MPT53, MPT59 (Ag85B), MPT63, and MPT64 were detected by strong staining in all pulmonary infiltrates (Tables 3 and 4). The presence of antigens was primarily seen as intracellular granular staining in the cytoplasm of inflammatory cells (Figure 1). The stained cells were uniformly distributed in the lesions. MPT44 was found in a lower number of cells as compared to other antigens. Table 3 shows the number of positive cases for each antigen.

**Table 3:** **Immunohistochemical analysis of** ***in vivo*** **expression of various mycobacterial antigens in the formalin fixed tissues obtained from** ***Mycobacterium tuberculosis*** **-infected lungs and lymph nodes, and non-tuberculous controls**

	No. with positive results/total number (%)***		
	Multibacillary lung tissues**	Tuberculous lymphadenitis	Non-TB controls
**ZN staining**	3/3 (100)	0/17 (0)	0/12 (0)
PCR*	3/3 (100)	17/17 (100)	0/12 (0)
***Secreted antigens***			
**MPT32**	3/3 (100)	3/17 (18)	0/12 (0)
**MPT44**	1/3 (33)	4/14 (28)	6/12 (50)
**MPT46**	3/3 (100)	0/17 (0)	1/12 (8)
**MPT51**	3/3 (100)	0/17 (0)	0/12 (0)
**MPT53**	3/3 (100)	3/17 (18)	1/12 (8)
**MPT59**	3/3 (100)	6/17 (35)	3/12 (25)
**MPT63**	3/3 (100)	2/17 (12)	2/12 (17)
**MPT64**	3/3 (100)	17/17 (100)	2/12 (17)
***Somatic antigens***			
**Mce1A**	3/3 (100)	17/17 (100)	8/12 (67)
**Hsp65**	3/3 (100)	17/17 (100)	12/12 (100)
**MPT57**	3/3 (100)	16/16 (100)	12/12 (100)

*PCR, N-PCR for amplification of IS6110.

**Vast number of bacilli were detectable by ZN staining.

***A case/control was labelled as positive if any positive signal was recorded in a lesion irrespective of the quality of stain.

**Table 4:** **Semiquantitative comparative analysis of the various mycobacterial antigens in the individual cases of pulmonary and lymph node tuberculous detected by immunohistochemistry done on formalin fixed paraffin embedded tissues**

	Secreted antigens								Somatic antigens		
	MPT32	MPT44	MPT46	MPT51	MPT53	MPT59	MPT63	MPT64	Mce1A	Hsp65	MPT57
**Lung 1**	+++++	-	+++++	+++++	+++++	++++	+++++	+++++	+++	+++++	++++
**Lung 2**	+++++	-	+++++	+++++	+++++	++++	+++++	+++++	+++	++	++
**Lung 3**	+++	+++	+++++	++++	++++	++++	++++	++++	++	++	+++
**LN 1**	-	-	-	-	-	SNC	-	+	+	+++	+++
**LN 2**	-	SNC	-	-	-	-	-	+	+++	+++, SNC	+++
**LN 3**	-	-	-	-	-	-	-	+	++	++++	++++
**LN 4**	-	-	-	-	-	-	-	+	+	++++	+
**LN 5**	-	-	-	-	-	-	-	+	+++, SNC	+++++	+++++
**LN 6**	+	-	-	-	-	SNC	-	++	++	++++	++++
**LN 7**	-	-	-	-	-	SNC	-	++	++	+++	+
**LN 8**	-	ND	-	-	SNC	SNC	-	++	+	+++	+++
**LN 9**	-	ND	-	-	-	-	-	++	++	+++	+++
**LN 10**	-	+	-	-	-	-	-	++	+	+++	++++
**LN 11**	-	ND	-	-	-	+	-	++	+	+++	+
**LN 12**	-	-	-	-	SNC	-	+	++	+++	+++	+++
**LN 13**	+	-	-	-	-	+	-	+++	+	+++++	+
**LN 14**	-	-	-	-	-	-	-	+++	+	+++	+++
**LN 15**	-	+	-	-	SNC	-	-	+++	+++	++++	++++
**LN 16**	+	SNC	-	-	-	-	+	+++	++	++	ND
**LN 17**	-	-	-	-	-	-	-	++	+	++	++++

LN: Lymph node, SNC; staining in the necrotic centres, ND; not determined.

+++++ ~ 80% positive granuloma cells, ++++ ~ 50% positive granuloma cells, +++ 10-25% positive granuloma cells, ++ 2-9% positive granuloma cells, + < 2% positive granuloma cells, - no staining.

### Secreted mycobacterial antigens in lymph nodes

**Figure 2:**
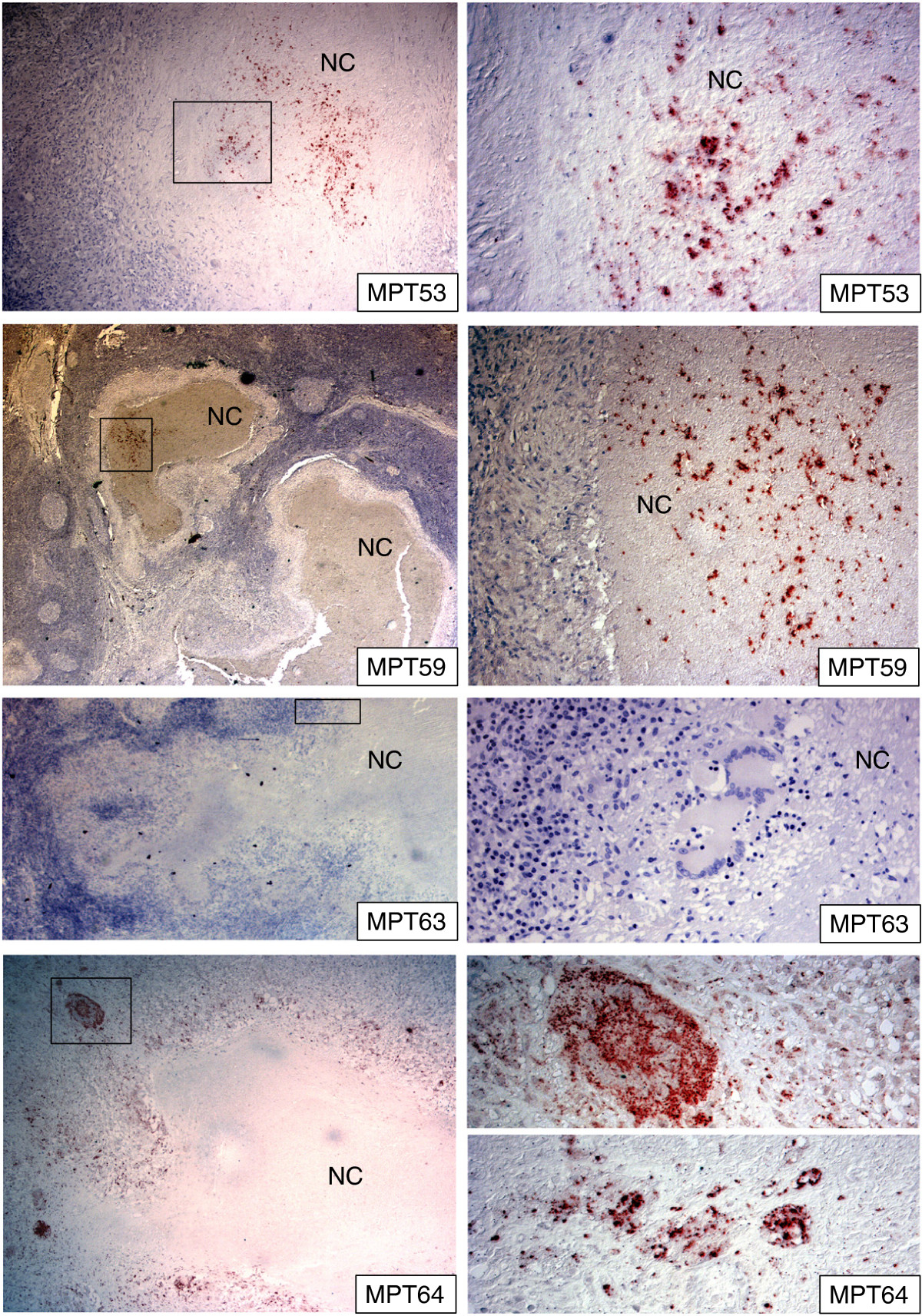
**Human lymph nodes tissue infected with** ***M. tuberculosis*** **showing the staining pattern of mycobacterial secreted antigens in the necrotic granulomas as detected by immunohistochemical staining.** Right column shows higher magnification of the marked areas in the corresponding pictures. There is a differential staining of various secreted antigens. MPT64 is detected in epithelioid cells and multinucleated giant cells, while the necrotic centres do not contain this antigen. MPT59 (Ag85B) and MPT63 were detected in few cases and were located only in the necrotic centres in these cases. Granuloma cells were not stained by these antibodies. The majority of the lymphadenitis granulomas were not stained for secreted antigens. NC = necrotic centres.

All secreted antigens described above were detected at a low level, or were absent, in the tuberculous lymphadenitis cases. The exception was MPT64, which were observed in all of the lymphadenitis cases, with variable staining intensities (Tables 3 and 4, Figure 2). Components of the antigen 85 complex: MPT44 (Ag85A), MPT51 (Ag85D), MPT59 (Ag85B), which constitute a large proportion of *in vitro*-secreted antigens, were identified in only 8/17 of cases. Among the positive cases, staining was found in less than 2% of granuloma cells, or only in the necrotic centres - typically in one or two out of 5–7 granulomas in a lymph node section (Table 4, Figure 2). The staining pattern was granular with strong intensity and seen as discrete localised stained areas (Figure 2). Despite the homology between various components of the antigen 85 complex, their staining pattern differed in this tissue. MPT44 (Ag85A) was seen in 4 cases; it was detected in less than 2% of granuloma cells in two of the cases, and only in necrotic centres in the other 2 cases. MPT59 (Ag85B) was detected in the necrotic centres in 4 cases. There was no agreement between the cases where these antigens were found. MPT51 (Ag85D) was not detected in any of the investigated sections. MPT53 was only found in necrotic centres of granulomas in three cases, and the staining pattern was similar to that of MPT59 (Ag85B) (Figure 2). MPT63 and MPT32 were identified in 2 and 3 cases respectively in less than 2% of granuloma cells, while MPT46 was not found in any of the lymphadenitis cases. (Tables 3 and 4).

### Somatic mycobacterial antigens in lung tissues

**Figure 3:**
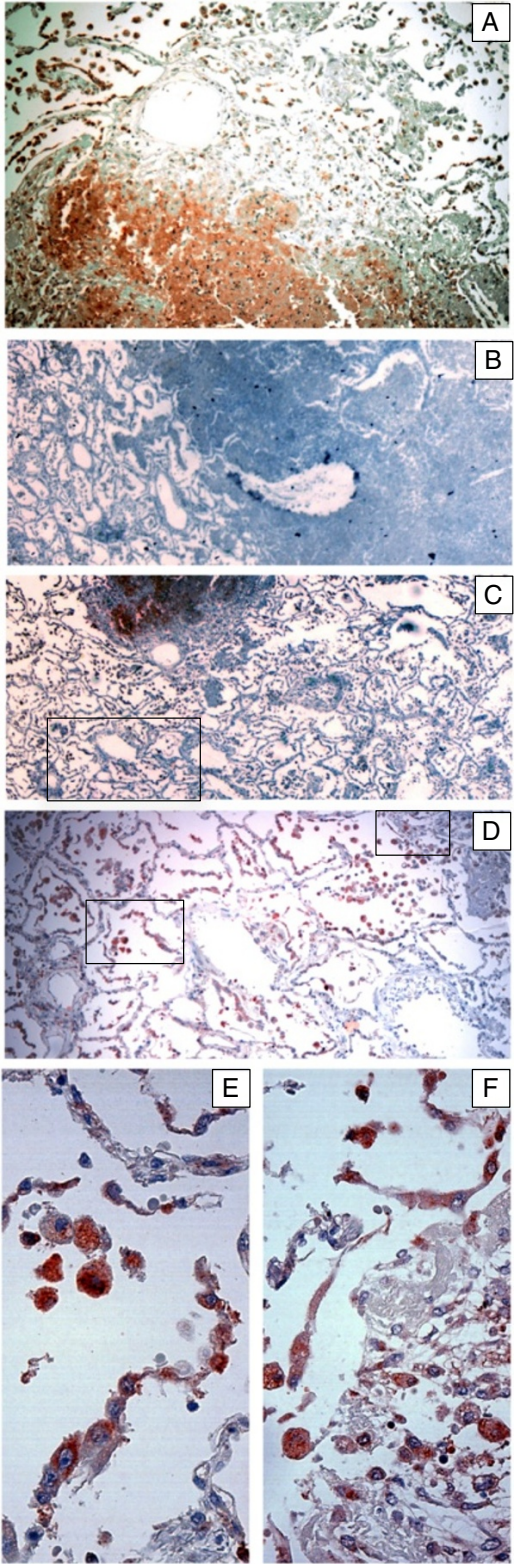
**Staining pattern of mycobacterial Mce1A antigen in human pulmonary tissues infected with** ***M. tuberculosis*** **as detected by immunohistochemical staining.** Panels **A-C** shows three different infiltrates from the same lung section, the area marked in **C** is shown in higher magnification in **D** and the two demarcated areas in panel D are magnified in panels **E-F**. The expression of Mce1A was relatively lower as compared to the other secreted antigens. One of the infiltrates did not contain this antigen, while the expression of other secreted antigens was abundant in this infiltrate (as shown in Figure 1). The other two infiltrates expressed this antigen at a relatively lower level as compared to the other secreted antigens. The lung parenchyma in vicinity of the infiltrates expressed this antigen more abundantly as compared to the other secreted antigens. Alveolar macrophages, activated macrophages, and alveolar lining cells expressed the antigen.

There was a difference in the staining patterns obtained using antibodies against somatic and secreted antigens. In the pulmonary lesions, a lower ratio of cells was stained for somatic antigens as compared to the secreted antigens and the intensity of staining was weaker as compared to the staining of the secreted antigens. In some areas, staining of anti-Mce1A would co-localise with that of the secreted antigens, while in other regions the anti-Mce1A staining was not detected despite the presence of a strong staining for all secreted antigens. Furthermore, cells stained by anti-Mce1A were observed in areas of the lungs without pneumonic infiltrates while secreted antigens were not stained in these areas (Figure 3).

### Somatic mycobacterial antigens in lymph node tissues

**Figure 4:**
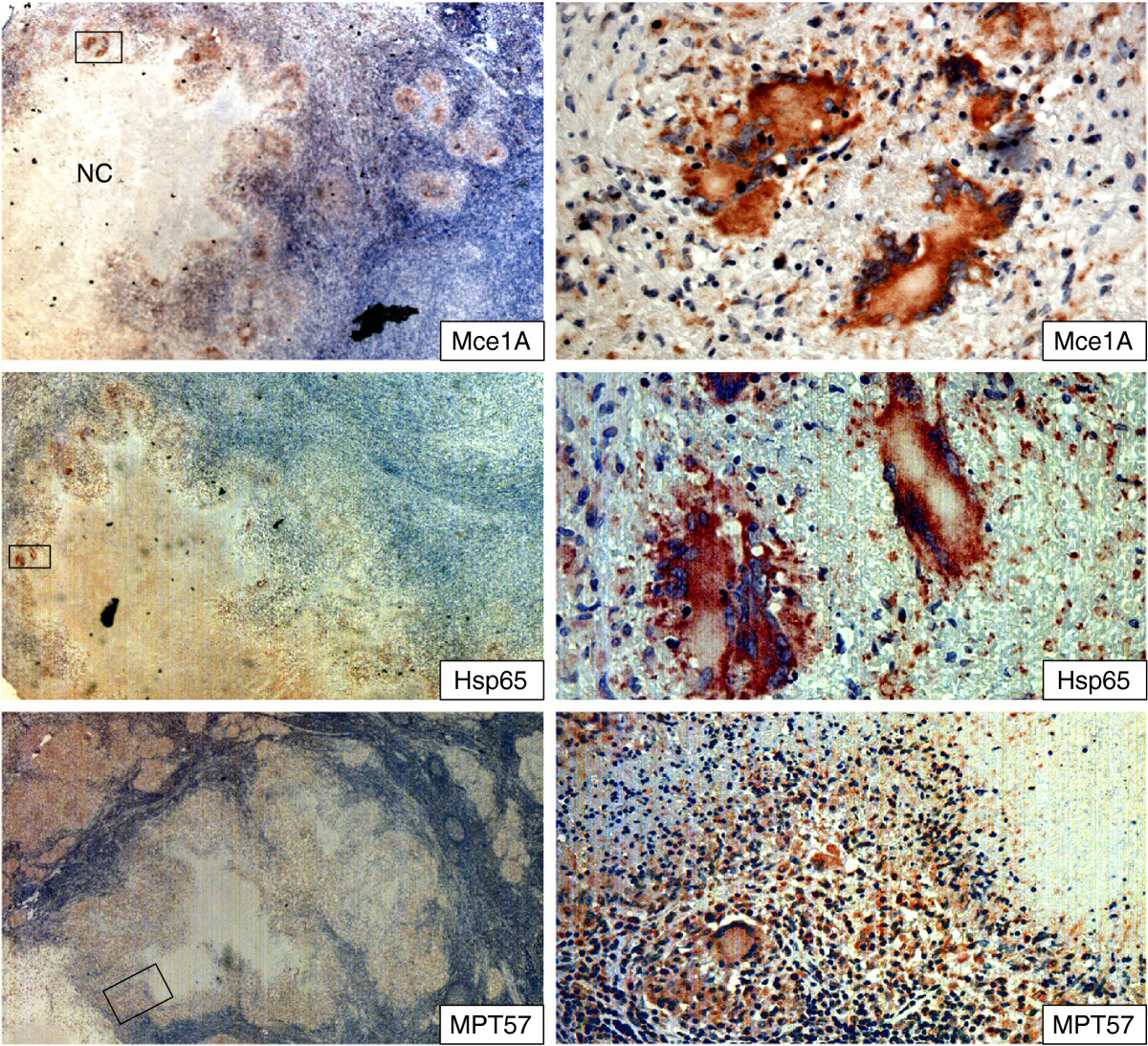
**Staining pattern of mycobacterial cell membrane (Mce1A) and somatic (MPT57 and Hsp65) antigens in lymph nodes tissues infected with** ***M. tuberculosis*** **, as detected by immunohistochemical staining.** The areas marked in the left column panels are shown in higher magnification in the right column panels. Mce1A and Hsp65 was stained strongly in epithelioid cells and multinucleated giant cells, while the staining intensity of MPT 57 was weaker in these cells. Mce1A was not detected in the necrotic centres. NC = necrotic centres.

In the tuberculous lymphadenitis granulomas, somatic antigens were detected in all the cases. Antigens were primarily found in the granuloma-, epithelioid- and multinucleated giant cells, and very little in necrotic centres (Figure 4). The staining was granular and found within the host cell cytoplasm with strong intensity. The number of stained cells varied among the cases as shown in Table 4. Hsp65 was detectable in a higher number of cells as compared to other antigens (Tables 3 and 4, Figure 4). The pattern of staining for MPT57 (GroES) was less granular and more diffuse than what was observed for the other two somatic antigens. In the granulomas, multinucleated giant cells were not stained as strongly by anti-MPT57 as they were by anti-Mce1A or anti-Hsp65 (Figure 4).

Among the negative controls, several cases also showed positive signals and they were recorded as positive, but here the intensity of staining was weaker and the staining pattern was diffuse rather than granular. Furthermore, the staining found in non-inflammatory cells was located in the nucleus and not in the cytoplasm, and it was primarily observed in the periphery of the section. Based on these differences in the quality and location of staining pattern the positive antigen staining in the negative controls was concluded as non-specific and it was possible to discriminate that by an expert pathologist. However this information of non-specific staining as positive signals is included in the results in order to highlight that which of the antigens/antibodies is better suited for use as a diagnostic test, where the interpretation should be more robust.

## Discussion

In this study, we analysed the expression of mycobacterial antigens in infected tissues.

One interesting observation was the differential staining pattern of secreted antigens in the lungs and lymph node lesions. While these antigens were found in high amounts in the pulmonary lesions, their level of staining was very low, or absent, in the lymph node granulomas. This contrasted the findings for somatic antigens, which were found with the same ratio in both types of lesions. The pulmonary lesions exhibited pneumonic infiltrates without proper necrotic granuloma formation, while the lymph node lesions had organised necrotic granulomas. Granuloma formation is known to be a correlate of an effective immune response resulting in control of bacillary multiplication and containment of infection. Relative absence of secreted mycobacterial antigens in the well-formed granulomas indicate that immune response is effective to bring bacillary multiplication under control, while the presence of somatic antigens imply their involvement in the persistence of infection.

We could not detect bacilli by acid-fast staining in the lymph node tissues, which might indicate that the number of intact bacilli is below a detectable level. However, it has been shown that mycobacteria tend to lose of their acid-fastness as they adapt to the stressful intracellular milieu within a granuloma [26–28]. This can provide an alternative explanation to why no bacilli could be detected in the lymph nodes using this technique. The mycobacterial response to stressful conditions is multifactorial. Other observed change is the down-regulation of a number of secreted antigens that are considered to be highly expressed during normal culture conditions, including Ag85A-C, MPT63 and MPT64 [29–31]. As the bacteria adapt to persistence and long-term survival within cells of the granuloma, many of the antigens required during, and immediately after, cell invasion, are no longer important for bacterial survival. This could explain why most of the investigated secreted antigens are nearly absent in the lymph nodes, while the somatic antigens were equally detected in both lung and lymph node tissues. Serodiagnostic studies have evaluated specificities of antibodies from sera of TB patients against different mycobacterial antigens including secreted-, membrane-, cell wall associated-, and cytosolic antigens. Although secreted antigens have been most commonly studied, it is the surface exposed components that appear to be most frequently recognised by patient sera [32, 33]. A study that described a lack of host immune response towards the secreted mycobacterial antigen MPT53 in humans and guinea pigs [34], lends support to the theory of a reduced presence of secreted antigens *in vivo* during stable disease. The question remains why the major secreted antigens in some cases were only detected in the necrotic centres and not in the granuloma cells. One possible explanation is that the discrete spots of staining in the necrotic centres represent cells that harbour multiplying bacilli that produce secreted antigens, but that these cells are gradually removed by cell lysis and necrosis.

There was a notable exception to the almost complete absence of staining of secreted antigens in the lymphadenitis tissues; the antigen MPT64 was identified in all granulomas. The explanation for this observation could be that MPT64 is not cleared away from the tissues and therefore accumulates in the granulomas over time. Structural analyses of this protein, suggest that it harbours a β-grasp motif that can form strong protein-protein interactions [35, 36]. We have previously studied the staining pattern of MPT64 in tuberculous pleural biopsies in HIV co-infected TB patients, where only about half of the biopsies had organised granulomatous inflammation. MPT64 antigen was more frequently detected in the granulomas than in the non-granulomatous inflammation (93% vs. 64%), while mycobacterial DNA was detected more frequently in the non-granulomatous inflammation as compared to granulomas [21]. This antigen therefore seem to preferentially accumulate within granuloma structures, even though the cells of the granuloma are efficiently restricting bacterial growth. The persistence of MPT64 in the tissues suggests a role in bacterial pathogenesis.

In pulmonary lesions without proper granuloma formation, the ratio of cells that stained positive for the somatic antigen Mce1A was lower, and the staining intensity was weaker, compared to the staining pattern of secreted antigens. Furthermore, the staining pattern of Mce1A and secreted antigens did not always overlap; certain regions were not stained by anti-Mce1A, despite being highly stained by antibodies against secreted antigens. In lymph nodes with organised granulomas, Mce1A was detected in all cases, located mainly intracellularly in the granuloma cells rather than necrotic centres as seen with some secreted antigens. The products of the *mce1* operon have been shown to be important for granuloma formation [37–39]. In this study a differential staining pattern of Mce1A in lungs with excessive pathology, but without the typical organised granulomas as compared to lymph node tissue, lend support to the theory of an involvement of this antigen in the formation of organised and protective granulomas.

## Conclusions

This study shows that major secreted mycobacterial antigens were found in high amounts in the pulmonary lesions that exhibited pneumonic infiltrates without proper necrotic granuloma formation, while their level of staining was very low, or absent, in the lymph node TB lesions with organised necrotic granulomas and very low bacillary load. One exception was MPT64, which was detected in both pulmonary and lymph node lesions. This indicates a preferential accumulation of this antigen within granuloma structures even if the cells of the granuloma can efficiently restrict bacterial growth, and suggests a role in the persistence of chronic infection. All three somatic antigens were detectable in both pulmonary and lymphadenitis lesions, and their expression pattern was different from that of the secreted antigens. The findings in this study have implications for the development of new diagnostic tools.

## Authors’ original submitted files for images

Below are the links to the authors’ original submitted files for images. Authors’ original file for figure 1 Authors’ original file for figure 2 Authors’ original file for figure 3 Authors’ original file for figure 4
