# Supplementary figures and images for: Differential in vivo expression of mycobacterial antigens in Mycobacterium tuberculosisinfected lungs and lymph node tissues
Source: BMC Infect Dis. 2014 Oct 3;14:535. doi: 10.1186/1471-2334-14-535 (PMC4287340; doi:10.1186/1471-2334-14-535)

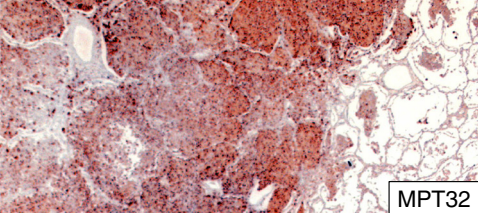

MPT32

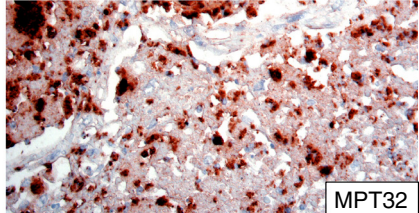

MPT32

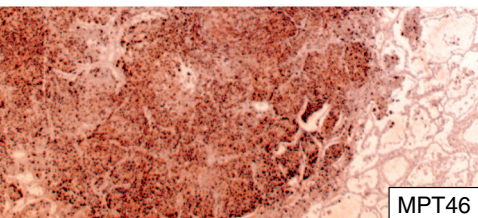

MPT46

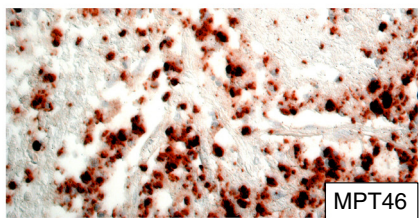

MPT46

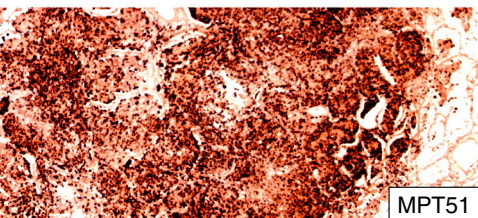

MPT51

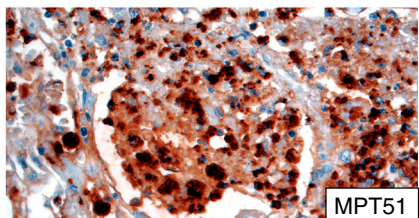

MPT51

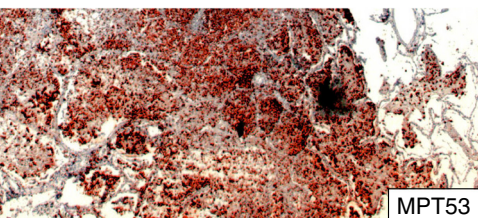

MPT53

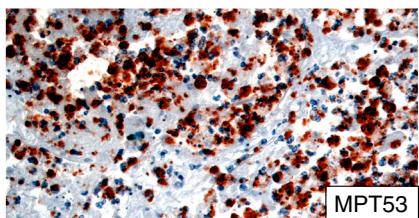

MPT53

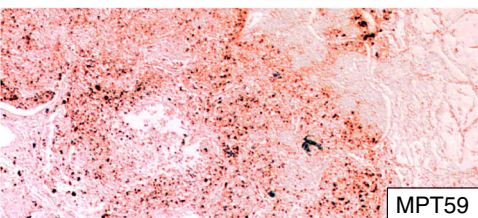

MPT59

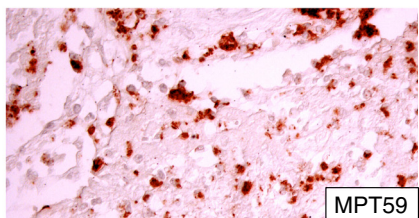

MPT59

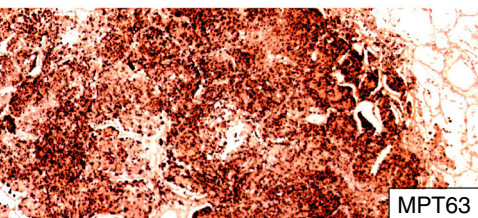

MPT63

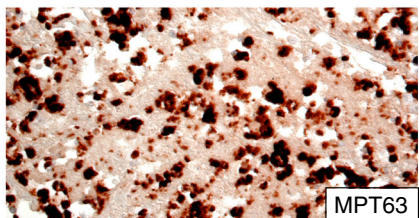

MPT63

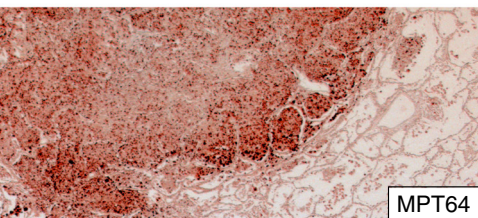

MPT64

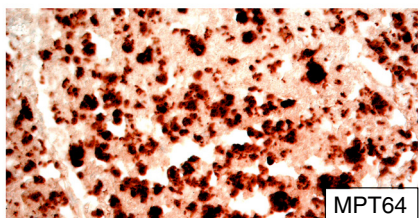

MPT64

Supplement: Supplementary file 1 — Authors’ original file for figure 1 [file 12879_2014_3851_MOESM1_ESM.pdf]

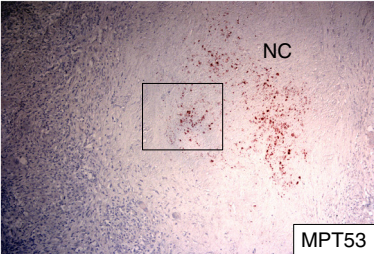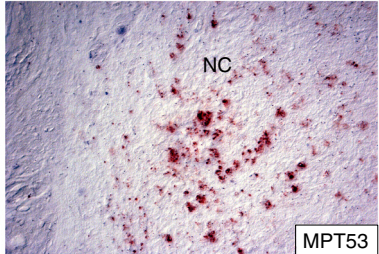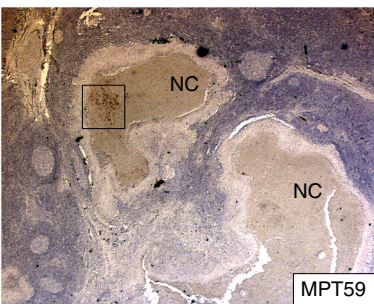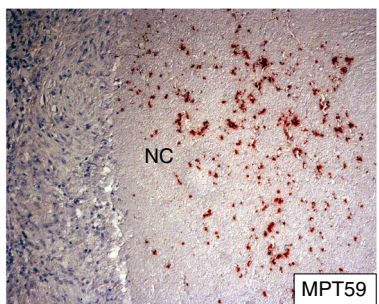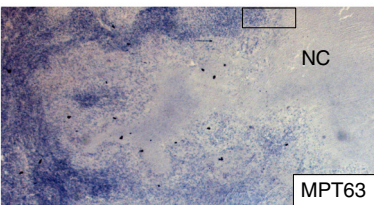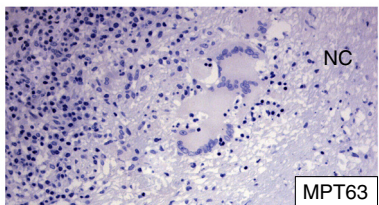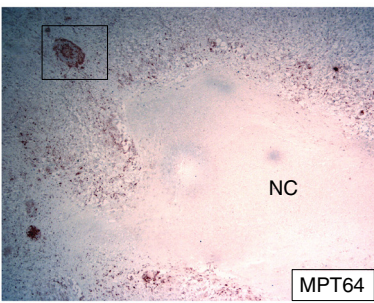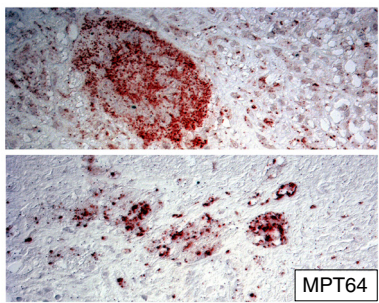

Supplement: Supplementary file 2 — Authors’ original file for figure 2 [file 12879_2014_3851_MOESM2_ESM.pdf]

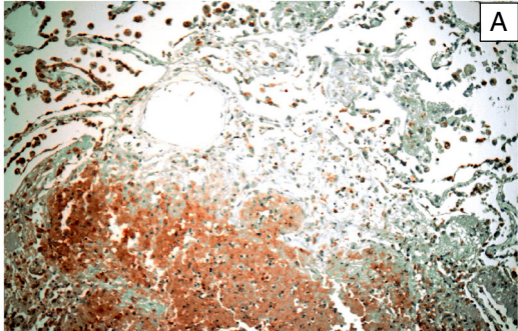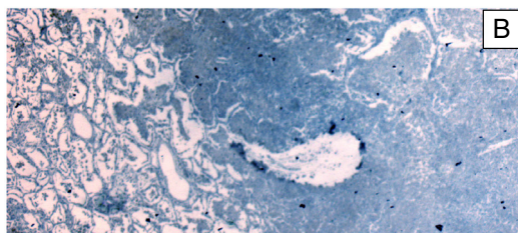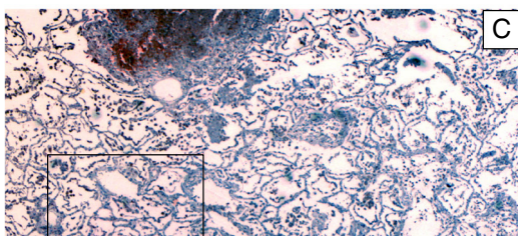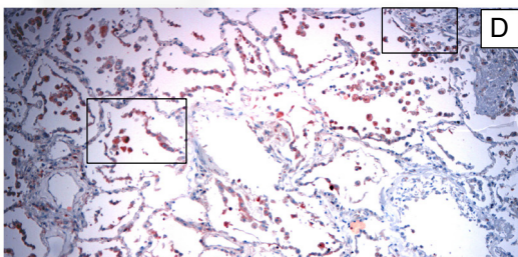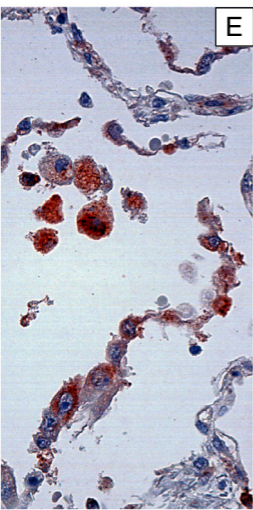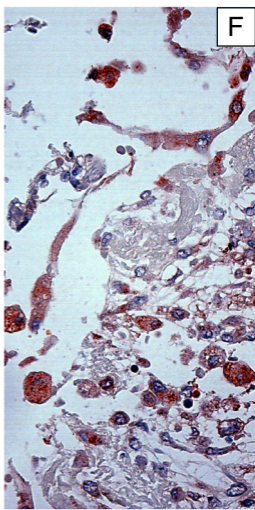

Supplement: Supplementary file 3 — Authors’ original file for figure 3 [file 12879_2014_3851_MOESM3_ESM.pdf]

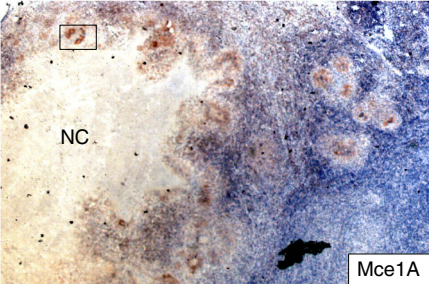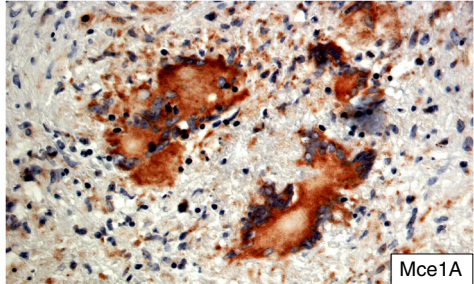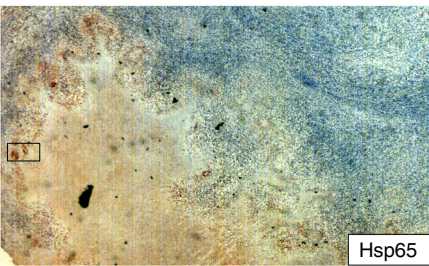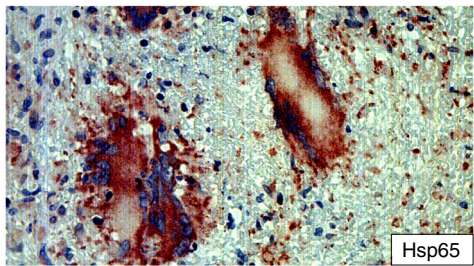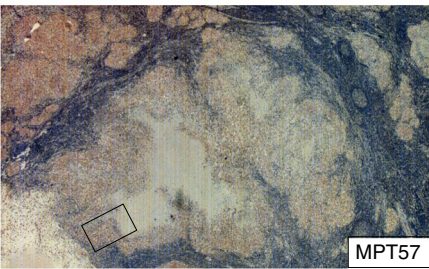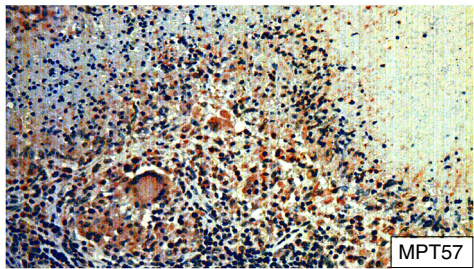

Supplement: Supplementary file 4 — Authors’ original file for figure 4 [file 12879_2014_3851_MOESM4_ESM.pdf]
